# Network analysis of the interrelationships among stigma, resilience, and self-esteem in lung cancer patients

**DOI:** 10.3389/fpsyg.2026.1952417

**Published:** 2026-09-30

**Authors:** Jinwen Hu, Jiao Yang, Yanqiu Zhao, Ruojia Wu, Nan Shen, Zhuma LuRong, Shu Chen, Qianrong Yang

**Affiliations:** 1 School of Nursing, Kunming Medical University, Kunming, China; 2 Nursing Department, Yunnan Cancer Hospital, Kunming, China; 3 Department of Thoracic Surgery II, Yunnan Cancer Hospital, Kunming, China; 4 Cancer Hospital Chinese Academy of Medical Sciences, Shenzhen Center, Shenzhen, China; 5 Nursing Department, Diqing Tibetan Autonomous Prefecture People’s Hospital, Shangri-La City, China

**Keywords:** lung cancer, network analysis, psychological resilience, self esteem, stigma

## Abstract

Stigma is a significant psychosocial challenge for patients with lung cancer and is closely related to psychological resilience and self-esteem. However, most existing studies have examined these factors in isolation, overlooking the complex interrelationships among their specific dimensions. This cross-sectional network analysis investigated 248 patients with lung cancer using validated measures of stigma, resilience, and self-esteem. A regularized partial correlation network comprising 11 dimensions was estimated using the EBICglasso algorithm. Expected Influence (EI) and Bridge Expected Influence (BEI) were used to assess overall and cross-community connectivity, respectively. “Hope for the Future” showed the highest observed EI (EI = 1.91), closely followed by “Support and Coping,” while “Self-Negation” showed the highest observed BEI (BEI = 0.517). These findings characterize the interconnected structure of stigma, resilience, and self-esteem and highlight future-oriented hope and negative self-evaluation as potentially relevant dimensions for psychosocial assessment and intervention development, although their roles should be further examined in longitudinal studies.

## 1 Introduction

Lung cancer is one of the most common cancers worldwide. According to a report by the International Agency for Research on Cancer (IARC) (Bray et al., 2024), approximately 20 million new cancer cases were diagnosed globally in 2022, with lung cancer accounting for nearly 2.5 million cases and causing 1.8 million deaths. In recent years, with advances in lung cancer treatment and improvements in patient survival rates, quality of life and well-being among patients with lung cancer have become a focus of medical research (Choudhury, 2023). However, patients diagnosed with lung cancer often develop pessimistic emotions due to long-term treatment, and may even exhibit psychological responses such as reluctance toward treatment or anticipatory grief (Morrison et al., 2017).

Within this context, stigma is an important concept for understanding the psychological experiences of patients with lung cancer. Lung cancer stigma encompasses negative social and psychological experiences, including perceived blame, judgment, rejection, and discrimination, as well as internalized feelings of shame, guilt, and self-blame (Hamann, 2018; McCann and Dunne, 2026). As a behavioral manifestation of stigma, discrimination may involve unfair or differential treatment and may arise in interactions with friends, family members, healthcare professionals, or society more broadly (Criswell et al., 2016). Lung cancer may be particularly susceptible to stigma because of its strong public association with cigarette smoking. Although smoking is the dominant risk factor for lung cancer, not all lung cancer cases occur among individuals with a history of smoking. Importantly, recent evidence indicates that lung cancer stigma is experienced regardless of smoking status (McCann and Dunne, 2026). Because smoking is commonly perceived as a controllable behavior, the association between smoking and lung cancer may foster assumptions of personal responsibility and blame, thereby contributing to both externalized and internalized stigma. Lung cancer stigma has been associated with a range of adverse psychological, behavioral, and social outcomes. Internalized stigma and self-blame are associated with lower self-worth and self-esteem, as well as greater feelings of helplessness, anxiety, and depression (Criswell et al., 2016; Pan, 2025). Lung cancer stigma may also make patients reluctant to disclose their diagnosis, particularly when they anticipate judgment or blame related to smoking (Criswell et al., 2016). Stigma and related feelings of shame and guilt may further contribute to delays in or avoidance of seeking help for symptom management and other healthcare needs (Filchner et al., 2022). Such delays in help-seeking may have implications for timely diagnosis and clinical outcomes. Of particular concern, stigmatizing attitudes may also occur within healthcare settings, where patients may perceive blame, differential treatment, or inadequate care from healthcare professionals (Criswell et al., 2016; Mcfadden et al., 2025). Recent qualitative evidence further suggests that the effects of lung cancer stigma may extend beyond patients themselves, with family members experiencing stigma by association through judgment from others and adverse social experiences (McCann and Dunne, 2026). Taken together, these findings suggest that lung cancer stigma is a multidimensional experience associated with substantial psychological, behavioral, and social consequences for patients. Identifying psychological resources associated with stigma in patients with lung cancer is therefore important for informing supportive interventions.

In particular, psychological resilience and self-esteem have been identified as two key positive resources that enhance patients’ psychological adaptation in coping with stigma (Liu and Zhong, 2020; Li, 2025). Psychological resilience is not only a crucial capacity for adapting to stress or adversity but also a dynamic psychological process (Rutter, 2000; Connor and Davidson, 2003). Research confirms that psychological resilience serves as a significant mediator in the psychological adjustment of lung cancer patients, mitigating the negative impact of stigma on self-perceived burden and quality of life, while also moderating overall well-being (Xu and Gao, 2025; Cho and Ryu, 2021). However, these studies have primarily focused on whether an association exists between stigma and resilience, without deeply explaining how they interact or systematically analyzing the roles of different resilience dimensions.

On the other hand, research also indicates that self-esteem level is a protective factor against stigma in cancer patients, which is defined as an individual’s positive evaluation and cognition of their overall worth in psychology (Fleming and Watts, 1980). Current research on the role of self-esteem in the psychosocial adaptation of cancer patients has largely focused on breast cancer populations, particularly those experiencing body image changes following mastectomy (Tang et al., 2023), for many young breast cancer patients, stigma is also a significant factor impairing self-esteem (Gao et al., 2024); Furthermore, studies on cervical cancer patients have found that stigma often obstructs marital and family happiness (Wang, 2025). These studies collectively suggest that maintaining self-esteem is one of the core needs for cancer patients coping with stigma. However, the relationship between stigma and self-esteem in the lung cancer population requires further exploration. Whether feelings of shame and guilt in lung cancer patients are exacerbated by lower self-esteem remains unclear.

In brief, stigma in lung cancer patients, as a negative emotional experience, not only intensifies emotional distress and persistently damages self-worth and increases guilt but may also further erode self-esteem. Psychological resilience, as an intrinsic positive psychological resource, may buffer the negative impact of stigma on self-concept by strengthening self-acceptance and identity, thereby helping to maintain self-esteem. Although existing studies have separately examined the pairwise relationships among resilience, self-esteem, and stigma in cancer patients, the complex interactive mechanisms among these three constructs have not been fully elucidated. Therefore, this study employs network analysis to construct a systemic network model of stigma, resilience, and self-esteem to reveal their internal connections and interaction pathways.

Network analysis is a method grounded in mathematical graph theory, constructing networks through “nodes” and “edges” to represent complex interrelationships among variables or dimensions (Hevey, 2018). This approach not only visualizes patterns of associations among nodes within a system but also allows the identification of dimensions with relatively high centrality or cross-community connectivity. From a positive psychology perspective, such information may help identify potentially intervention-relevant psychological dimensions and generate hypotheses for the development and evaluation of targeted psychosocial interventions for patients with lung cancer.

## 2 Materials and methods

### 2.1 Participants and sampling

From June 2023 to June 2024, this study employed a convenience sampling method to recruit 248 lung cancer patients from the department of thoracic surgery at the third affiliated hospital of Kunming Medical University as participants.

Inclusion criteria were: (1) patients diagnosed with lung cancer and aware of their diagnosis; (2) providing informed consent and being conscious. Exclusion criteria were: (1) presence of mental illnesses; (2) presence of severe cognitive or communication impairments that would preclude completion of the study assessments. According to network analysis sample size requirements (Epskamp, 2012), The sample size should exceed the total number of parameters, where total parameters equal threshold parameters plus pairwise association parameters (Shang et al., 2024). Threshold parameters equal the number of nodes, and pairwise association parameters equal [nodes × (nodes − 1)]/2. This study constructed a network based on 11 dimensions from three scales, resulting in 11 nodes. Thus, threshold parameters = 11, and pairwise association parameters = (11 × 10)/2 = 55. Consequently, the minimum sample size was calculated as 66 cases. However, to ensure model stability, it is recommended to include 3 to 5 participants per parameter. Therefore, the minimum sample size was 198 cases. The final sample size of this study was 248 cases, meeting the network analysis sample size requirement. The study was approved by the Ethics Review Committee of Kunming Medical University (Ethics No.: KMMU2021MEC117).

### 2.2 Measures

#### 2.2.1 General information questionnaire

A general information questionnaire was used to collect participants’ demographic and health-related variables, including age, gender, ethnicity, education level, residence, smoking status, and treatment modality.

#### 2.2.2 The self-esteem scale

The Self-Esteem Scale, developed by Rosenberg (1965), assesses an individual’s sense of self-worth and self-acceptance. This study used the Chinese version translated and introduced by Ji and Yu (1999). This self-report scale comprises 10 items covering two dimensions: Self-Negation and Self-Affirmation. It employs a 4-point Likert scale (1 = strongly disagree to 4 = strongly agree). The total score ranges from 10 to 40, with higher scores indicating higher self-esteem. The Cronbach’s α coefficient for this scale in the present study was 0.82.

#### 2.2.3 The lung cancer stigma scale

The Lung Cancer Stigma Scale was developed by Cataldo (2011) to assess perceived stigma among patients with lung cancer. This study used the Chinese version translated and validated by Yuan et al. (2017). This self-report scale consists of 31 items covering four dimensions: shame, social isolation, discrimination, and smoking-related stigma. It uses a 4-point Likert scale (1 = strongly disagree to 4 = strongly agree). The total score ranges from 31 to 124, with higher scores indicating greater perceived stigma. The Cronbach’s α coefficient of the Chinese version was 0.93, compared with 0.97 in the present study.

#### 2.2.4 The resilience scale specific to cancer

The Resilience Scale Specific to Cancer (RS-SC), developed and validated by Ye et al. (2018), is a 25-item self-report instrument specifically designed to assess resilience in Chinese adults with cancer. This scale comprises 25 items covering five dimensions: Generic Elements, Benefit Finding, Support and Coping, Hope for the Future, and Meaning for Existence. It uses a 5-point Likert scale (1 = strongly disagree to 5 = strongly agree). The total score ranges from 25 to 125, with higher scores indicating greater resilience. Although the RS-SC is less widely used than generic resilience measures, it was selected for the present study because it was specifically developed and validated for Chinese patients with cancer and was designed to capture cancer-specific aspects of resilience that may not be fully represented by generic resilience instruments. Importantly, patients with lung cancer were included in the original validation sample, supporting the relevance of the RS-SC to the population examined in the present study (Ye et al., 2018). The original RS-SC demonstrated sound psychometric properties, with a Cronbach’s α of 0.83 and a test–retest reliability of 0.87. The Cronbach’s α coefficient in the present study was 0.97.

### 2.3 Statistical analysis

All network analyses in this study were conducted using the open-source software R (version 4.5.1).

#### 2.3.1 Descriptive statistics

Normally distributed continuous variables were presented as means ± standard deviations, while categorical variables were presented as frequencies and percentages. Independent-samples t-tests were used for between-group comparisons. Cronbach’s α coefficients were calculated for all scales to assess their internal consistency.

#### 2.3.2 Network estimation

Following the reporting standards for cross-sectional network analysis (Burger et al., 2023), the network was estimated using the bootnet package and visualized using the qgraph package in R (Epskamp, 2012). A regularized partial correlation network was estimated using the EBICglasso algorithm, which applies LASSO regularization to obtain a sparse network. Network estimation was based on Spearman correlations, and the EBIC hyperparameter γ was set to 0.50. The Fruchterman–Reingold force-directed algorithm was used for network visualization. The network was constructed at the dimension level rather than the individual-item level. The 11 nodes represented four dimensions of stigma, five dimensions of resilience, and two dimensions of self-esteem. These dimensions were not selected using a correlation-based cutoff; rather, all 11 dimension scores were included as nodes in the network. Edges represented conditional associations between pairs of nodes after controlling for all other nodes in the network.

#### 2.3.3 Centrality

Expected Influence (EI) was selected as the primary centrality index rather than strength centrality because EI retains the signs of edge weights and therefore accounts for both positive and negative associations. EI was calculated as the sum of the signed edge weights connecting a given node to all other nodes in the network, with higher EI values indicating stronger overall signed connectivity. Closeness and betweenness were not emphasized because these path-based indices rely on assumptions regarding shortest paths and information flow that may be difficult to interpret in psychological networks (Costantini et al., 2015; Robinaugh, 2016).

Bridge Expected Influence (BEI; 1-step) was used to assess cross-community connectivity among the three predefined communities of stigma, resilience, and self-esteem (Jones et al., 2021). BEI was calculated as the sum of the signed edge weights directly connecting a given node to nodes in the other communities. BEI was selected rather than bridge strength because BEI retains the signs of cross-community edge weights and therefore distinguishes positive from negative cross-community associations (Epskamp, 2018). Higher BEI values indicate stronger overall signed connectivity with nodes belonging to other psychological communities.

#### 2.3.4 Accuracy, stability, and bootstrap difference tests

Network accuracy and stability were assessed using bootstrap procedures implemented in the bootnet package with 1,000 resamples (Epskamp, 2018). The accuracy of edge-weight estimates was assessed using non-parametric bootstrapped 95% confidence intervals (CIs), with narrower CIs indicating greater precision. The stability of EI and BEI estimates was assessed using case-dropping bootstrap procedures and quantified using the correlation stability (CS) coefficient. A CS coefficient above 0.50 was considered indicative of good stability, whereas values below 0.25 were considered undesirable (Epskamp, 2018). Bootstrapped pairwise difference tests were additionally conducted for edge weights, EI, and BEI. A difference was considered statistically significant when the bootstrapped 95% CI for the difference between two estimates did not include zero.

## 3 Results

### 3.1 Descriptive statistics

This study included 248 patients with lung cancer. Sociodemographic characteristics are presented in Table 1. The descriptive statistics for stigma, resilience, and self-esteem are presented in Table 2.

**Table 1:** Sociodemographic and clinical characteristics of the participants.

Characteristic	Category	*n* (%)
Patient status at survey	Postoperative follow-up	52 (21.0)
	Postoperative inpatient	196 (79.0)
Age (years)	25–34	8 (3.2)
	35–44	24 (9.7)
	45–54	77 (31.0)
	55–64	96 (38.7)
	65–74	35 (14.1)
	75–80	8 (3.2)
	Mean ± SD	57.3 ± 10.6
Sex	Male	113 (45.6)
	Female	135 (54.4)
Ethnicity	Han	214(86.3)
	Ethnic minority	34(13.7)
Marital status	Unmarried	1 (0.4)
	Married	237 (95.6)
	Divorced	3 (1.2)
	Widowed	7 (2.8)
Residence	Rural	110 (44.4)
	Urban	138 (55.6)
Education level	Primary school or below	72 (29.0)
	Junior high school	76 (30.6)
	High school/secondary school	35 (14.1)
	College	31 (12.5)
	Bachelor’s degree	31 (12.5)
	Master’s degree or above	3 (1.2)
Occupation	Farmer	90 (36.3)
	Worker	33 (13.3)
	Civil servant/teacher/military	27 (10.9)
	Freelancer	26 (10.5)
	Healthcare worker	10 (4.0)
	Unemployed	22 (8.9)
	Retired	37 (14.9)
	Other	3 (1.2)
Family relationships	Poor	1 (0.4)
	Fair	25 (10.1)
	Good	88 (35.5)
	Very good	134 (54.0)
Monthly household income (RMB/month)	< 1,000	34 (13.7)
	1,000–2,000	55 (22.2)
	2,001–4,999	90 (36.3)
	5,000–10,000	60 (24.2)
	> 10,000	9 (3.6)
Health insurance	Self-pay	9 (3.6)
	Provincial medical insurance	15 (6.0)
	Municipal medical insurance	55 (22.2)
	New Rural Cooperative Medical Scheme	78 (31.5)
	Urban resident medical insurance	84 (33.9)
	Commercial insurance	7 (2.8)
Comorbidities^1^	None	160 (64.5)
	Hypertension	37 (14.9)
	Diabetes	19 (7.7)
	Cardiovascular disease	5 (2.0)
	Unknown	27 (10.9)
Primary caregiver	Parents	10 (4.0)
	Spouse	125 (50.4)
	Children	95 (38.3)
	Siblings	10 (4.0)
	Caregiver/nanny	8 (3.2)
Caregiver health status	Good	191 (77.0)
	Fair	55 (22.2)
	Poor	2 (0.8)
Smoking status	Smoker	55 (22.2)
	Non-smoker	193 (77.8)
Treatment modality^2^	Surgery	203 (83.5)
	Chemotherapy	39 (16.0)
	Traditional Chinese Medicine	1 (0.4)

SD, standard deviation; RMB, Chinese renminbi.

^1^Comorbidities were reported as mutually exclusive categories (participants may have had only one comorbidity recorded per the survey structure); “Unknown” indicates participants who were unclear about their comorbidity status.

^2^For treatment modality, percentages were calculated based on 243 valid responses (5 missing) and may not sum to 100% because of rounding.

**Table 2:** Descriptive statistics of stigma, resilience, and self-esteem.

Variable	*N*	Mean ± SD
Stigma	248	96.92 ± 14.87
Resilience	248	96.08 ± 17.87
Self-Esteem	248	27.20 ± 1.81

### 3.2 Network analysis

#### 3.2.1 Network structure of stigma, resilience, and self-esteem

The combined network of stigma, resilience, and self-esteem in lung cancer patients, estimated using the EBICglasso method, along with node labels, is shown in Figure 1. The network comprises 11 nodes divided into three communities: stigma, resilience, and self-esteem. Of all possible 55 edges, 35 were non-zero (63.64%). The strongest positive connection within the network was between “Social Isolation” (CL2) and “Discrimination” (CL3) within the stigma community (edge weight = 0.59). The strongest negative connection was between “Self-Negation” (SE1) and “Self-Affirmation” (SE2) within the self-esteem community (edge weight = −0.51). A detailed edge-weight matrix is presented in Table 3.

**Figure 1:**
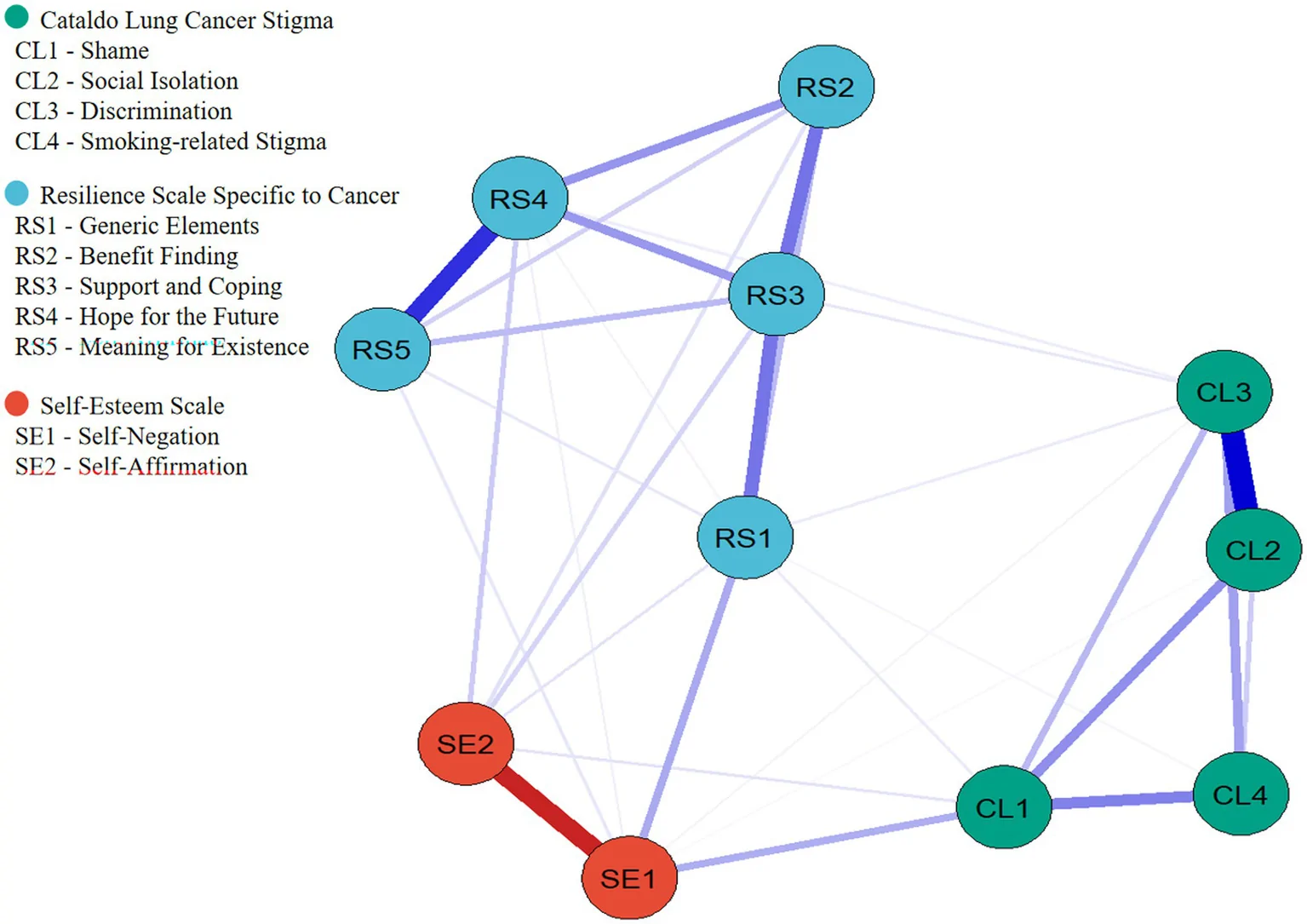
Network structure of stigma, resilience, and self-esteem in patients with lung cancer.

**Table 3:** Edge-weight matrix of the stigma, resilience, and self-esteem network.

Network nodes		Stigma				Resilience					Self-esteem	
		CL1	CL2	CL3	CL4	RS1	RS2	RS3	RS4	RS5	SE1	SE2
Stigma	CL1	0.00	0.26	0.16	0.29	0.05	0.00	0.00	0.00	0.00	0.19	0.07
	CL2	0.26	0.00	0.59	0.11	0.00	0.00	0.00	0.00	0.00	0.02	0.00
	CL3	0.16	0.59	0.00	0.22	0.05	0.00	−0.06	0.04	0.00	0.03	0.00
	CL4	0.29	0.11	0.22	0.00	0.03	0.00	0.00	0.00	0.00	0.00	0.00
Resilience	RS1	0.05	0.00	0.05	0.03	0.00	0.16	0.32	0.03	0.06	0.20	0.06
	RS2	0.00	0.00	0.00	0.00	0.16	0.00	0.32	0.24	0.11	0.00	0.07
	RS3	0.00	0.00	−0.06	0.00	0.32	0.32	0.00	0.24	0.16	0.00	0.10
	RS4	0.00	0.00	0.04	0.00	0.03	0.24	0.24	0.00	0.48	0.04	0.12
	RS5	0.00	0.00	0.00	0.00	0.06	0.11	0.16	0.48	0.00	0.05	0.00
Self-esteem	SE1	0.19	0.02	0.03	0.00	0.20	0.00	0.00	0.04	0.05	0.00	−0.51
	SE2	0.07	0.00	0.00	0.00	0.06	0.07	0.10	0.12	0.00	−0.51	0.00

Values represent regularized partial correlation edge weights estimated using EBICglasso. Positive and negative values indicate positive and negative conditional associations, respectively. Off-diagonal values of 0 indicate edges that were shrunk to zero by regularization. CL1, Shame; CL2, Social Isolation; CL3, Discrimination; CL4, Smoking-related Stigma; RS1, Generic Elements; RS2, Benefit Finding; RS3, Support and Coping; RS4, Hope for the Future; RS5, Meaning for Existence; SE1, Self-Negation; SE2, Self-Affirmation.

#### 3.2.2 Centrality

The Expected Influence (EI) centrality indices for the nodes in the stigma, resilience, and self-esteem network are shown in Figure 2. “Hope for the Future” (RS4, EI = 19.1) had the highest observed EI, closely followed by “Support and Coping” (RS3, EI = 1.186) and “Discrimination” (CL3, EI = 1.139). Overall, RS4, RS3, and CL3 showed relatively high signed connectivity within the network.

**Figure 2:**
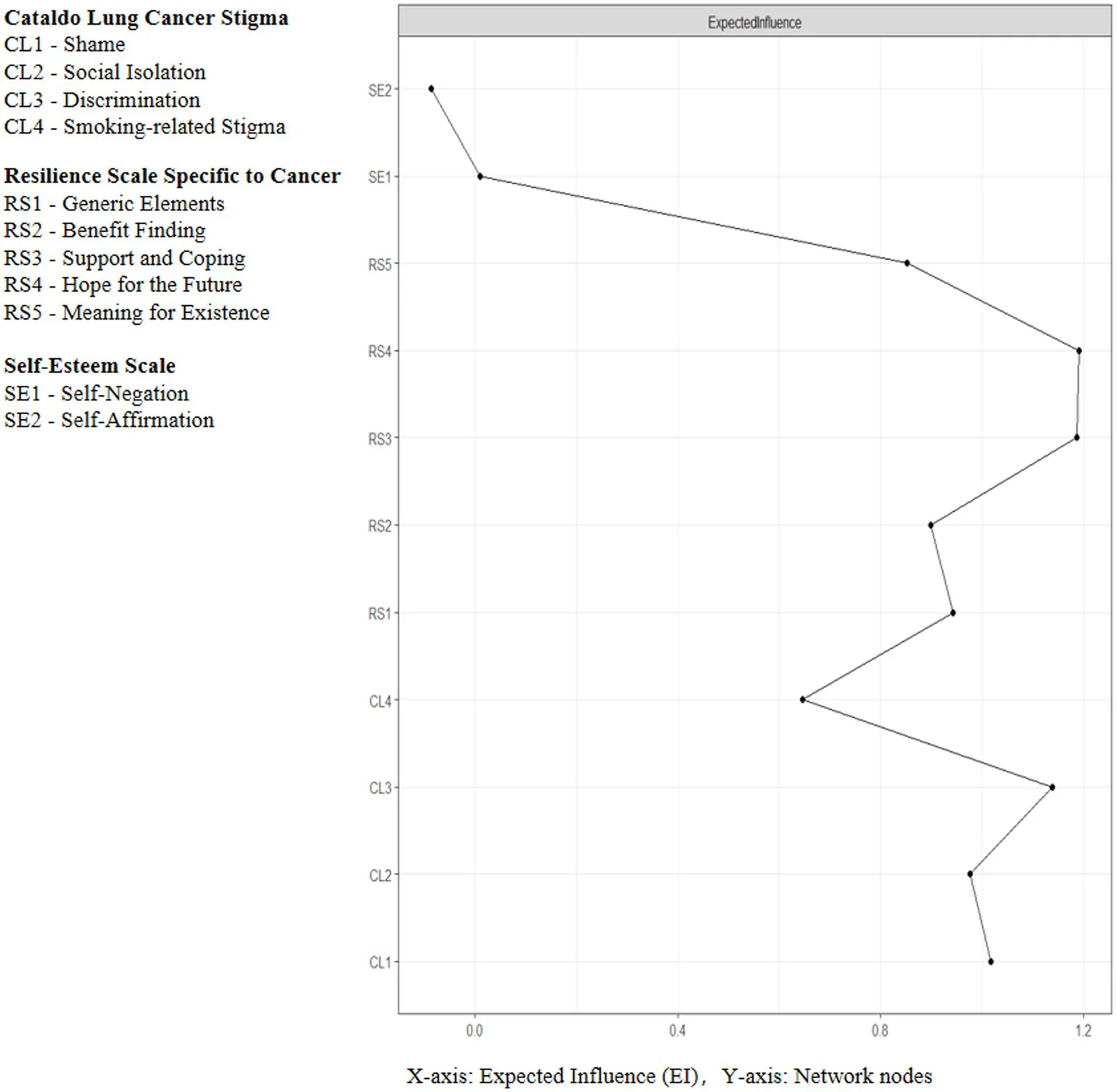
Expected Influence of nodes in the stigma, resilience, and self-esteem network. The *x*-axis represents the Expected Influence (EI) value, with higher values indicating stronger overall signed connectivity within the network; the *y*-axis represents the network nodes.

#### 3.2.3 Bridge centrality

The Bridge Expected Influence (BEI) analysis results are shown in Figure 3. “Self-Negation” (SE1, BEI = 0.517) showed the highest observed BEI, followed by “Self-Affirmation” (SE2, BEI = 0.420) and “Generic Elements” (RS1, BEI = 0.381). SE1 showed its strongest cross-community connections with RS1 (edge weight = 0.20) and “Shame” (CL1, edge weight = 0.19). In contrast, “Hope for the Future” (RS4), despite showing the highest observed EI, had a lower BEI (BEI = 0.20), indicating relatively lower cross-community connectivity.

**Figure 3:**
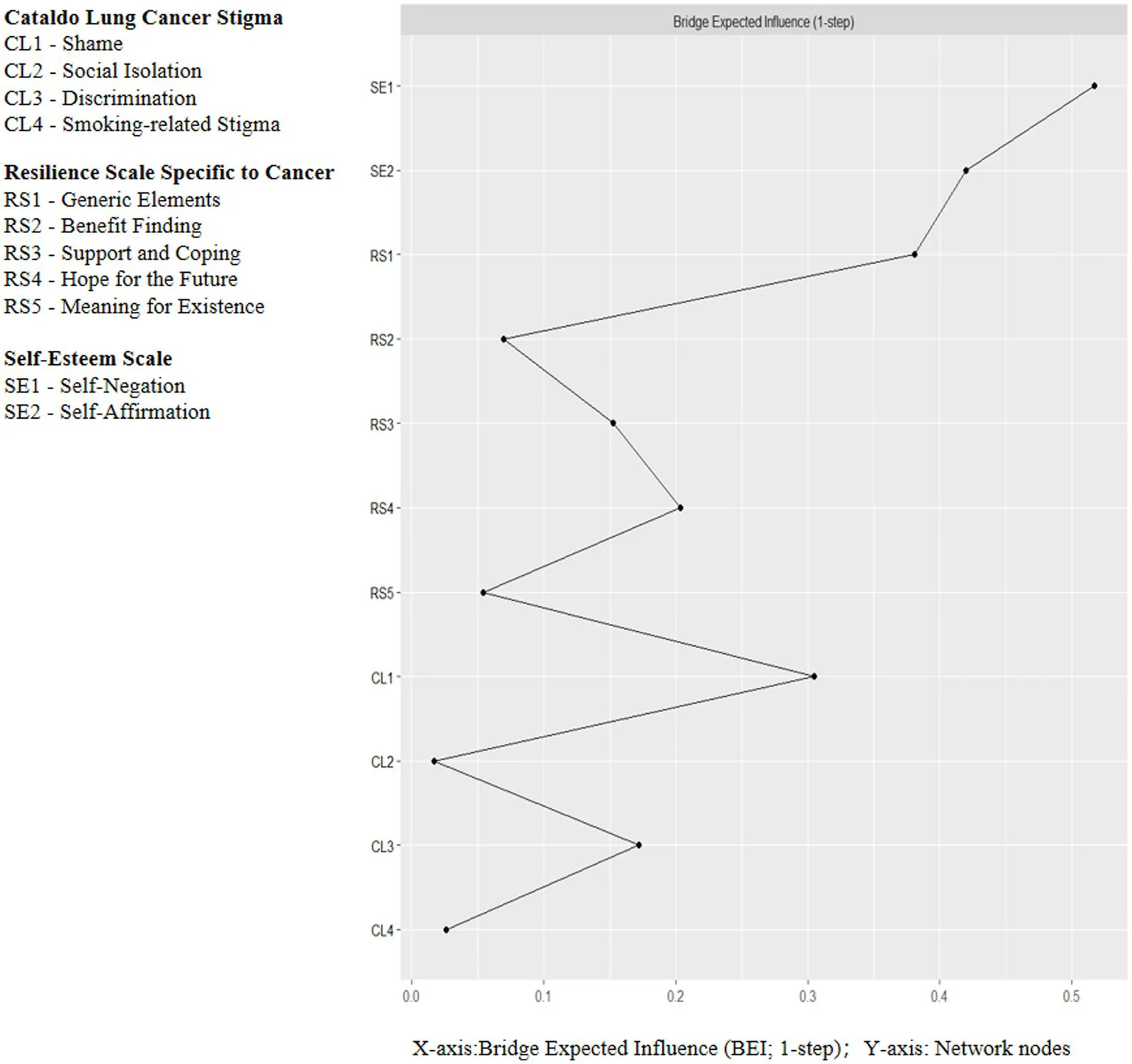
Bridge Expected Influence of nodes in the stigma, resilience, and self-esteem network. The *x*-axis represents the Bridge Expected Influence (BEI; 1-step) value, with higher values indicating stronger overall signed connectivity with nodes in other communities; the *y*-axis represents the network nodes.

#### 3.2.4 Accuracy and stability

Figure 4 presents the bootstrapped 95% confidence intervals (CIs) for the edge-weight estimates. The relatively narrow CIs for the stronger edges suggest acceptable accuracy of the corresponding edge-weight estimates.

**Figure 4:**
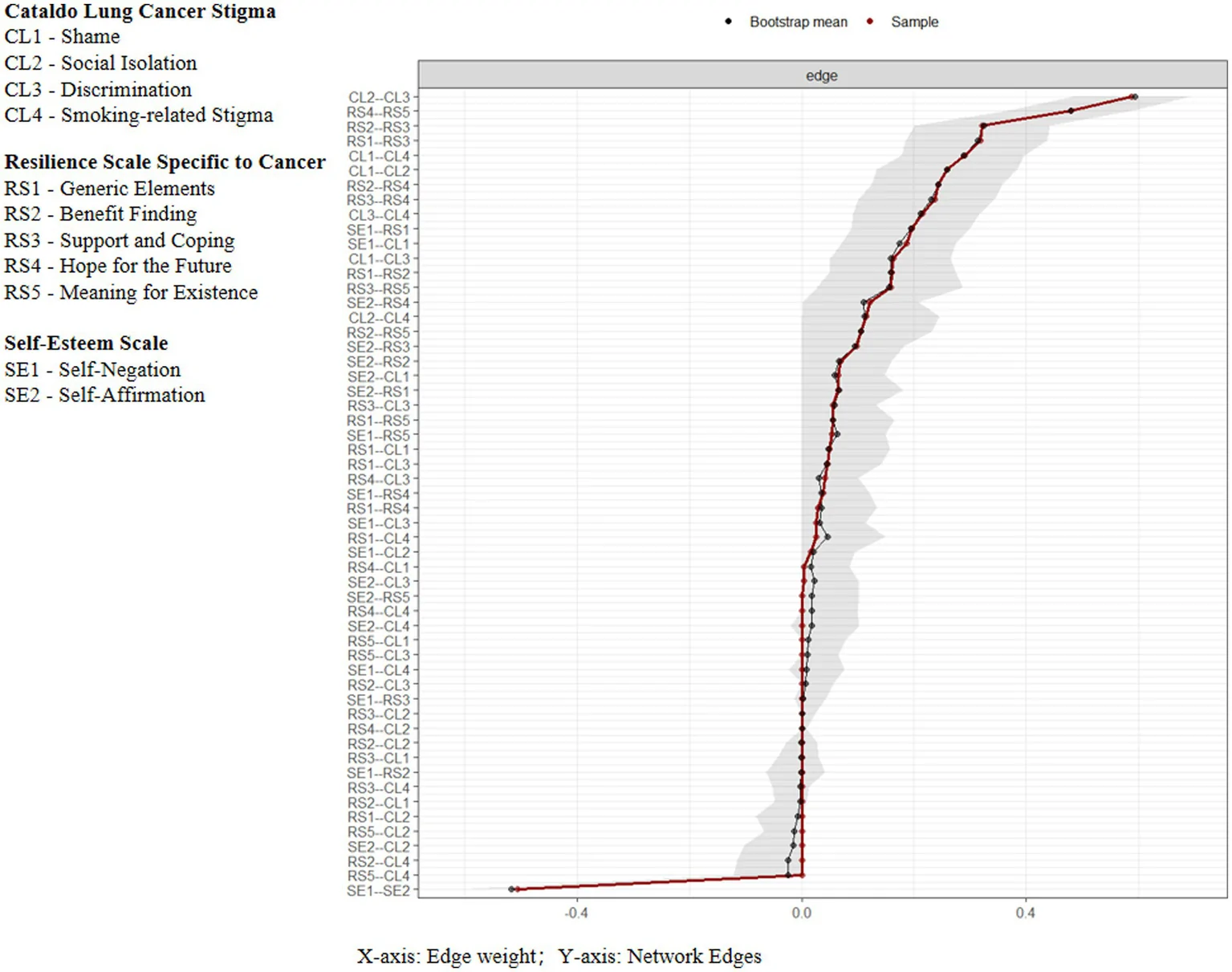
Non-parametric bootstrapped 95% confidence intervals for edge weights. The *x*-axis represents the estimated edge weights, and the *y*-axis represents the network edges. Black points indicate the bootstrap mean edge weights, red points indicate the edge weights estimated in the original sample, and the gray areas represent the bootstrapped 95% confidence intervals. Narrower confidence intervals indicate greater precision of the edge-weight estimates.

Figure 5 presents the stability results for EI and BEI. The correlation stability (CS) coefficients for EI and BEI were 0.75 and 0.67, respectively, both exceeding the recommended threshold of 0.50 and indicating good stability of the centrality estimates.

**Figure 5:**
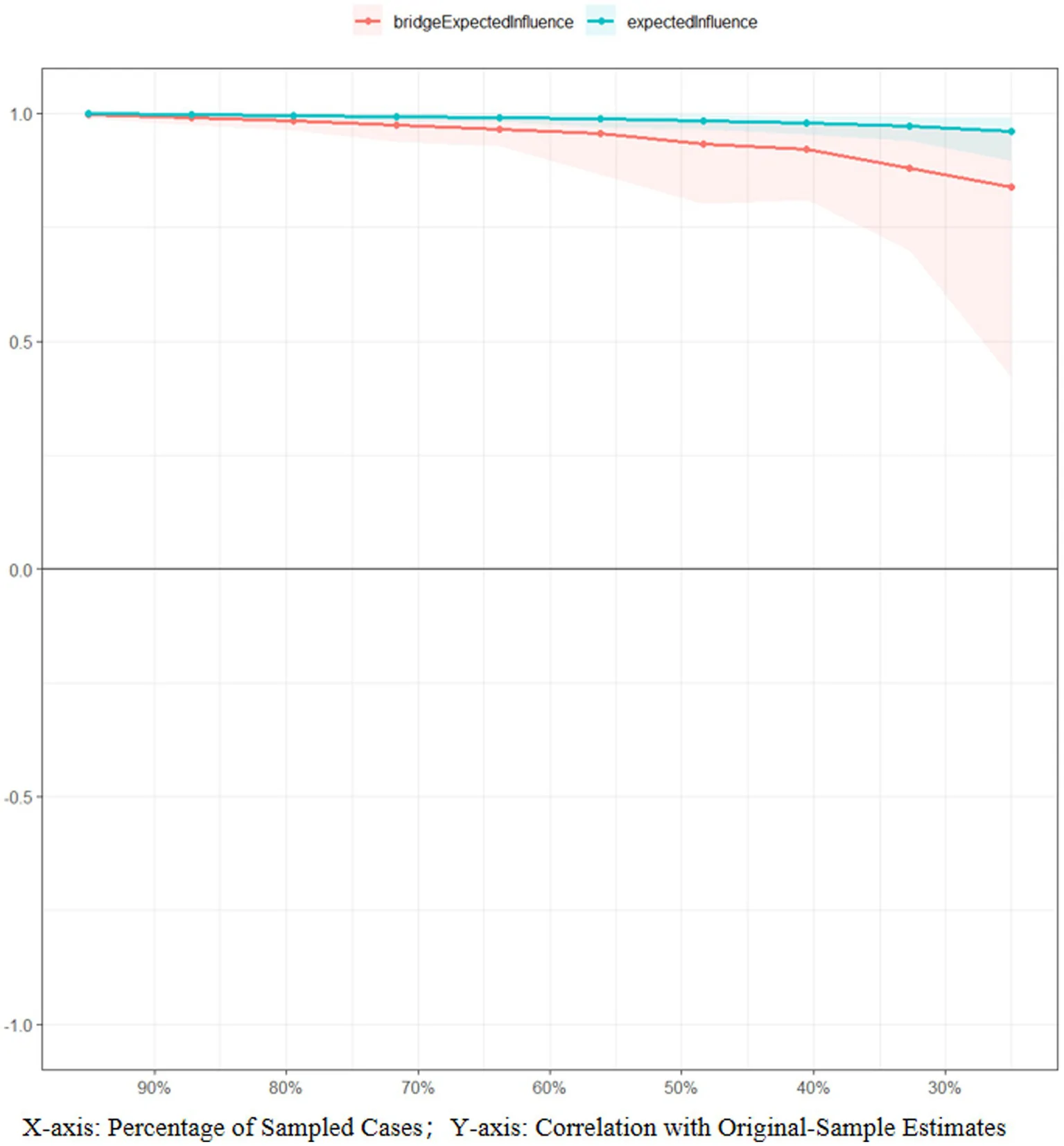
Case-dropping bootstrap stability of Expected Influence and Bridge Expected Influence. The *x*-axis represents the percentage of cases retained in the case-dropping bootstrap procedure, and the *y*-axis represents the average correlation between the centrality estimates obtained from the case-dropped samples and those obtained from the original sample. The lines represent Expected Influence (EI) and Bridge Expected Influence (BEI), with the shaded areas indicating the corresponding 95% confidence intervals.

#### 3.2.5 Bootstrap difference tests

Figure 6 presents the bootstrapped pairwise difference tests among the non-zero edge weights. Several of the strongest edges differed significantly from many of the weaker edges, whereas differences among some of the higher-weight edges were not statistically significant.

**Figure 6:**
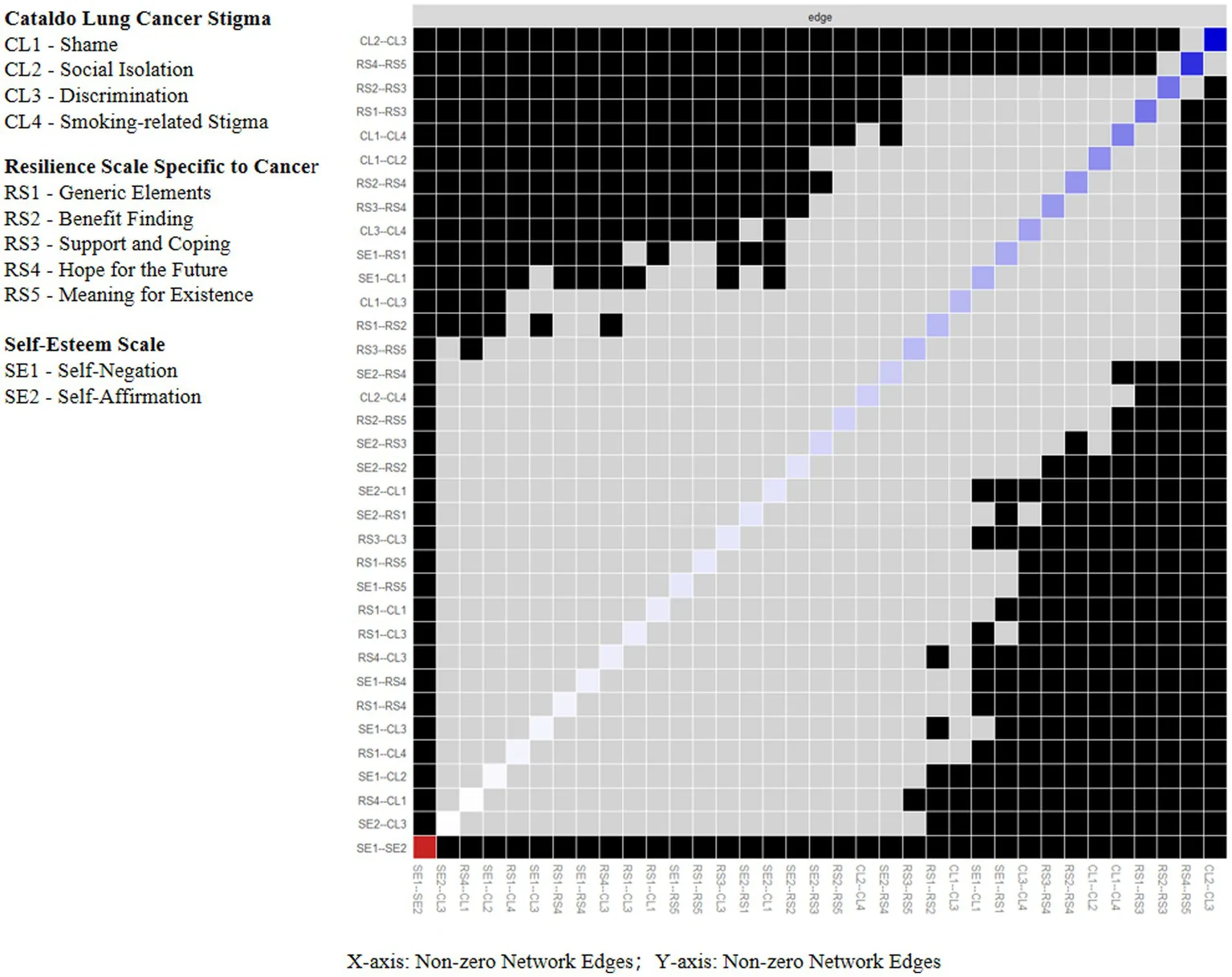
Bootstrapped pairwise difference tests for non-zero edge weights. The *x*- and *y*-axes represent the non-zero network edges being compared. Black cells indicate pairs of edge weights for which the bootstrapped 95% confidence interval of the difference did not include zero, whereas gray cells indicate non-significant differences. Colored cells along the diagonal represent the edge-weight estimates in the original sample.

Figure 7 presents the bootstrapped pairwise difference tests for EI. RS4 and RS3 showed the highest observed EI values, but the difference between them was not statistically significant. Their EI values also did not differ significantly from those of several other nodes with relatively high EI, although significant differences were observed compared with several nodes with lower EI values.

**Figure 7:**
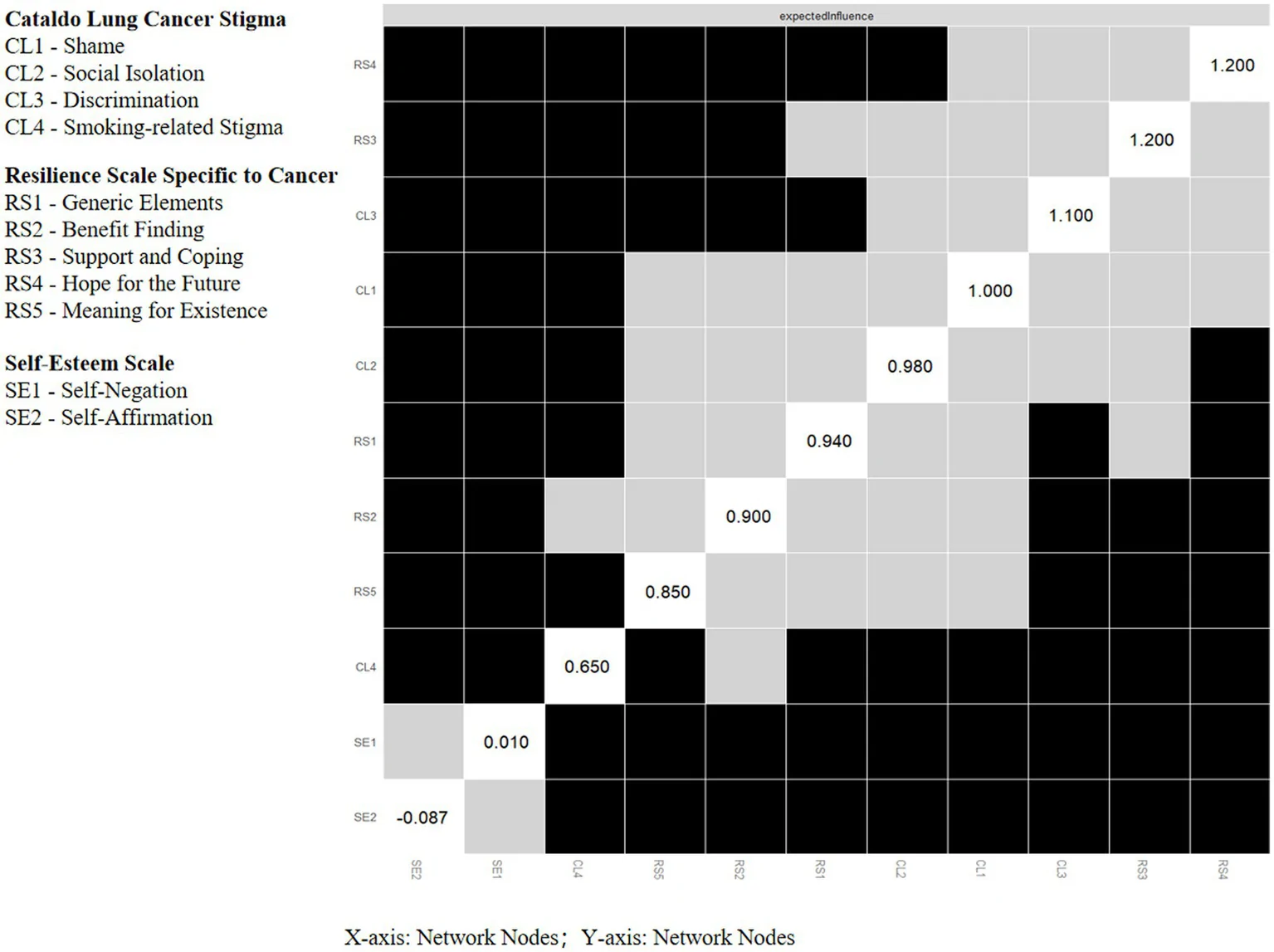
Bootstrapped pairwise difference tests for Expected Influence. The x- and y-axes represent the network nodes being compared. Black cells indicate pairs of nodes for which the bootstrapped 95% confidence interval of the difference in Expected Influence (EI) did not include zero, whereas gray cells indicate non-significant differences. Colored cells along the diagonal represent the EI estimates in the original sample.

Figure 8 presents the bootstrapped pairwise difference tests for BEI. SE1 showed the highest observed BEI, followed by SE2 and RS1. However, the BEI of SE1 did not differ significantly from those of SE2 and RS1, whereas SE1 differed significantly from several nodes with lower BEI values.

**Figure 8:**
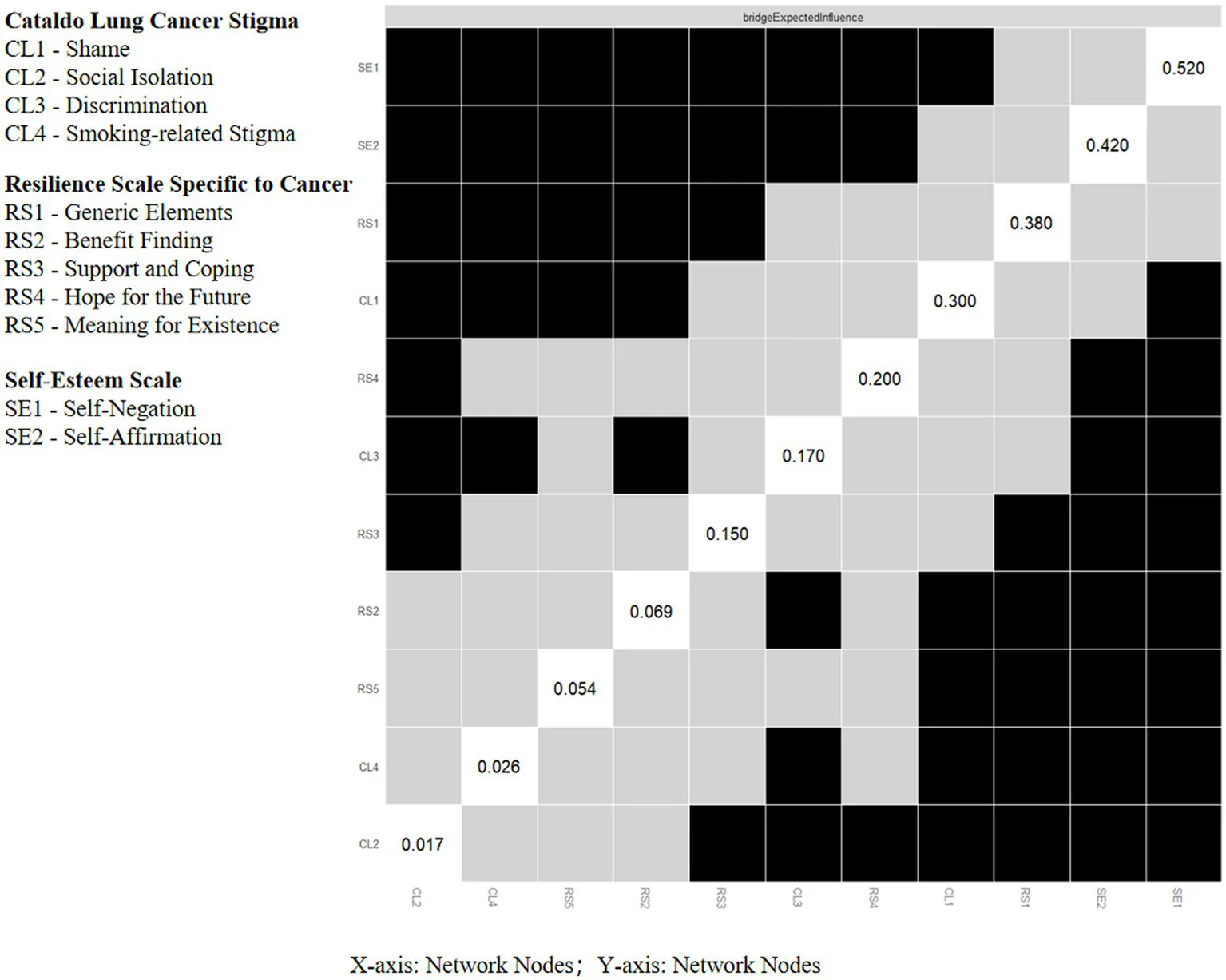
Bootstrapped pairwise difference tests for Bridge Expected Influence. The *x*- and *y*-axes represent the network nodes being compared. Black cells indicate pairs of nodes for which the bootstrapped 95% confidence interval of the difference in Bridge Expected Influence (BEI) did not include zero, whereas gray cells indicate non-significant differences. Colored cells along the diagonal represent the BEI estimates in the original sample.

## 4 Discussion

This study employed network analysis to explore the interrelationships among stigma, psychological resilience, and self-esteem in patients with lung cancer. The network analysis results indicated that “Hope for the Future,” a dimension of psychological resilience, showed the highest observed EI and was among the most central dimensions in the network. “Self-Negation,” a dimension of self-esteem, showed the highest observed BEI, indicating relatively high cross-community connectivity among stigma, resilience, and self-esteem. These findings preliminarily highlight potentially important dimensions and cross-community connections within the psychological network of stigma, resilience, and self-esteem in patients with lung cancer.

### 4.1 The current status of stigma among patients with lung cancer

The mean stigma score in the present study was 96.92 ± 14.87, suggesting a relatively high level of perceived stigma among the participants. This score was higher than that reported by Yu et al. (2022) (61.94 ± 12.48), who used the same Chinese version of the Lung Cancer Stigma Scale, but lower than that reported by Cataldo (2012) (102.6 ± 20.22). These differences across studies may reflect variations in sample characteristics, sociocultural contexts, and healthcare environments.

### 4.2 The central role of hope for the future in the stigma, resilience, and self-esteem network

The network analysis showed that “Hope for the Future” (RS4) had the highest observed Expected Influence (EI), suggesting that it was among the most central dimensions in the stigma, resilience, and self-esteem network. However, the bootstrap difference test showed that the EI of “Hope for the Future” (RS4)did not differ significantly from that of “Support and Coping” (RS3) or several other dimensions with relatively high EI values. Therefore, its highest observed EI should not be interpreted as evidence that “Hope for the Future” (RS4) was uniquely or significantly more central than all other dimensions. Nevertheless, its high observed EI suggests that future-oriented hope may represent an important psychological resource within the broader pattern of associations among resilience, stigma, and self-esteem.

This interpretation is consistent with Steffen’s research (Steffen, 2018), which identified hope as an important protective factor for social functioning in patients with lung cancer. Recent qualitative evidence from patients with terminal cancer further highlights hope as a dynamic and multifaceted psychological resource, through which individuals maintain a future-oriented perspective and engage in meaning-making while navigating illness-related uncertainty (Hayden et al., 2026). Gokler-Danisman’s model (Gokler-Danisman, 2017) also identified hope and stigma as protective and risk factors for social functioning, respectively. The present network further suggests that “Hope for the Future” does not exist in isolation but is embedded within a broader pattern of associations with other resilience dimensions, including “Meaning for Existence” and “Benefit Finding.”

One possible theoretical interpretation of these associations is provided by Fredrickson’s Broaden-and-Build Theory (Fredrickson, 2001). From this perspective, future-oriented positive emotions such as hope may broaden cognitive and coping repertoires and facilitate the development of psychological resources, potentially supporting meaning-making and adaptive responses to adversity. Taken together, these findings suggest that “Hope for the Future” may represent an important dimension within the broader psychological network rather than an isolated protective resource. Interventions aimed at fostering hope may therefore warrant further investigation as a potential approach to supporting psychological adaptation among patients with lung cancer. However, given the cross-sectional nature of the present study, longitudinal and intervention studies are needed to determine whether changes in hope lead to subsequent changes in stigma or self-esteem.

### 4.3 The bridging role of self-negation in the stigma, resilience, and self-esteem network

“Self-Negation” (SE1) showed the highest observed Bridge Expected Influence (BEI), suggesting that it was among the dimensions with relatively strong cross-community connectivity in the stigma, resilience, and self-esteem network. However, the bootstrap difference test indicated that its BEI did not differ significantly from those of “Self-Affirmation” (SE2) and “Generic Elements” (RS1). Therefore, the highest observed BEI of “Self-Negation” should not be interpreted as evidence that it was uniquely or significantly more important as a bridge than all other dimensions. Nevertheless, its relatively high BEI suggests that negative self-evaluation may be particularly relevant to the pattern of associations between self-esteem and the other psychological domains examined in this study.

This interpretation is broadly consistent with previous research showing an association between lower self-esteem and greater internalized stigma, although some of this evidence has been derived from populations with psychiatric disorders rather than cancer (Nabors et al., 2014). Within the present network, “Self-Negation” reflects a negative evaluation of overall self-worth, providing a conceptually plausible link between experiences of stigma and self-evaluative processes. The stigma-induced identity threat framework may also provide a useful perspective for interpreting this finding. Previous research applying this framework in the context of lung cancer has suggested that stigma may threaten patients’ social identity and self-worth and has documented associations between lung cancer stigma, lower self-esteem, and psychosocial distress (Maggio, 2015).

Taken together, the relatively high BEI of “Self-Negation” suggests that negative self-evaluation may be an important dimension linking self-esteem with stigma and resilience within the observed network. However, because the present network is cross-sectional, the direction of these associations cannot be determined. Longitudinal studies are therefore needed to examine whether changes in self-negation precede or follow changes in stigma, resilience, and other aspects of self-esteem.

### 4.4 Practical implications

The present findings provide several potential directions for the development of psychosocial support for patients with lung cancer. Given the relatively high observed centrality of “Hope for the Future,” clinicians could consider assessing patients’ future-oriented expectations and sources of hope as part of psychosocial assessment. For patients reporting diminished hope, intervention developers could consider incorporating hope-supportive components, such as helping patients identify personally meaningful and achievable goals and develop strategies for pursuing these goals. A recent single-arm trial of the “Pathways” hope-enhancing intervention among patients undergoing treatment for advanced lung cancer demonstrated promising feasibility and acceptability, together with preliminary improvements in hope and cancer-related goal interference (McLouth et al., 2024). In addition, a positive psychological intervention based on the PERMA framework has been associated with improved hope among patients receiving chemotherapy for lung cancer (Tu, 2021). Evidence that patients may prefer psychological support during active treatment also provides a potential opportunity for integrating such approaches into routine care (McLouth et al., 2021).

The relatively high observed BEI of “Self-Negation” also suggests that negative self-evaluation may warrant attention in psychosocial assessment and intervention development. Clinicians could consider assessing pronounced negative self-evaluations among patients experiencing stigma, while intervention developers could examine whether cognitive-behavioral approaches aimed at identifying and reframing maladaptive self-evaluations are acceptable and beneficial in patients with lung cancer. Although evidence specific to lung cancer remains limited, CBT-based approaches incorporating self-esteem enhancement have shown beneficial effects on self-esteem and quality of life in other cancer populations (Rat, 2025). Rather than treating hope and self-negation as isolated intervention targets, future studies could evaluate whether interventions addressing both future-oriented psychological resources and negative self-evaluation are feasible and provide additional benefit.

At the healthcare service and policy levels, these findings support greater attention to stigma-sensitive psychosocial care for patients with lung cancer. Healthcare services could consider integrating assessment of stigma, resilience-related needs, and self-esteem into supportive care pathways and providing healthcare professionals with training to recognize and avoid stigmatizing communication. Policymakers and service planners could also support the development and evaluation of accessible psychosocial services alongside routine lung cancer care. Importantly, however, the network findings identify potentially intervention-relevant dimensions rather than established causal intervention targets. Prospective longitudinal and intervention studies are required before it can be concluded that modifying hope or self-negation leads to subsequent reductions in stigma or improvements in psychosocial adaptation.

### 4.5 Limitations

This study used a cross-sectional design. Although this approach is useful for characterizing the conditional association patterns among stigma, resilience, and self-esteem in patients with lung cancer, it does not permit conclusions regarding temporal ordering or causal relationships among the dimensions. Therefore, the centrality and bridge centrality findings should not be interpreted as evidence that the identified dimensions causally influence other components of the network. Future longitudinal network studies are needed to examine the temporal dynamics of these relationships and determine whether changes in highly central or cross-community dimensions precede changes in other dimensions. Additionally, although efforts were made to include eligible participants, obtaining the sample faced certain challenges due to the sensitivity of the psychological topics involved and limited awareness of their own condition among some patients during data collection, based on ethical considerations and research feasibility. Consequently, the final sample size is relatively limited. This may, to some extent, restrict the stability and generalizability of the findings. Future research, under feasible conditions, could expand the sample size and include patients from more diverse cultural and medical backgrounds to enhance the universality of the conclusions.

## 5 Conclusion

This cross-sectional network study of 248 patients with lung cancer characterized the interrelationships among 11 dimensions of stigma, resilience, and self-esteem. “Hope for the Future” showed the highest observed EI and was among the dimensions with relatively high overall connectivity, whereas “Self-Negation” showed the highest observed BEI and relatively strong cross-community connectivity. Bootstrap difference tests, however, indicated that neither dimension was uniquely more central or bridging than all other dimensions with relatively high values. These findings highlight future-oriented hope and negative self-evaluation as potentially relevant dimensions for further investigation in the psychosocial care of patients with lung cancer. Clinicians and intervention developers may consider these dimensions when developing and evaluating supportive approaches, while healthcare services may benefit from greater integration of stigma-sensitive psychosocial assessment and support into routine lung cancer care. However, the cross-sectional design precludes conclusions regarding temporal or causal relationships. Longitudinal and intervention studies are needed to determine whether changes in these dimensions precede changes elsewhere in the network and whether targeting them can improve stigma-related and psychosocial outcomes.

## Data Availability

The raw data supporting the conclusions of this article will be made available by the authors, without undue reservation.
